# Correlation Between Demographic Attributes and Awareness Levels for the Early Detection of Breast Cancer in Jeddah, Saudi Arabia

**DOI:** 10.7759/cureus.115657

**Published:** 2026-09-02

**Authors:** Ali Elsarraj M Abdelmageed, Taysir A Wedatalla, Baqer A Almohammedali, Abdullah W Abumadian, Abdullah M Abdo

**Affiliations:** 1 Surgery, Ibn Sina National College for Medical Studies, Jeddah, SAU; 2 Pathology, Ribat University Hospital, Khartoum, SDN; 3 Surgery, Ibn Sina College Hospital, Jeddah, SAU

**Keywords:** awareness, breast cancer, demographic attributes, early detection, jeddah saudi arabia

## Abstract

Background: To reduce breast cancer incidence in Saudi Arabia, research, education, and public health measures should prioritize early detection. This study aimed to assess breast cancer symptoms and early-detection awareness among women aged ≥35 years recruited in Jeddah and to examine its association with age, educational attainment, marital status, and employment status.

Methodology: This descriptive cross-sectional survey was conducted in Jeddah, Saudi Arabia, from June 1, 2024, to May 30, 2025. Local female residents of Jeddah were conveniently selected as contributors. Females who resided in Jeddah for at least six months were included in the study. Participants with suspected or confirmed breast cancer and those who did not meet the three main inclusion criteria (female, age 35 or older, and Jeddah residency for six months) were excluded. A purpose-designed questionnaire was developed, validated, and subsequently used for data collection. Statistical analysis was performed using the software of IBM SPSS Statistics Version 22 (IBM Corp., Armonk, NY, USA). A total of 369 valid responses were included in the final analysis.

Results: The analysis showed a major gap in early breast cancer detection awareness, with 255 (69.1%) of our respondents scoring poorly. Possession of a college degree or higher education was found to correlate significantly with moderate awareness levels (P-value = 0.004, adjusted odds ratio = 2.55 at a confidence interval of 95%, upper boundary = 4.86, lower boundary = 1.34, the coefficient B/standard error ratio = 2.80), while stable marriage showed a significant correlation with high awareness levels (P-value = 0.03, adjusted odds ratio = 3.3 at a confidence interval of 95%, upper boundary = 9.72, lower boundary = 1.125, the coefficient B/standard error ratio = 2.17). Awareness was not statistically determined by age group or employment status.

Conclusion: Awareness of breast cancer symptoms and early detection procedures is inadequate among female residents of Jeddah. Awareness of breast cancer symptoms in this cohort positively correlated with higher educational attainment and stable marital status. No statistically significant correlation was found between age group or employment status and the level of awareness of breast cancer symptoms. Early detection of breast cancer in Jeddah should prioritize age-susceptible women with low education. It is recommended that future investigations evaluate these demographic characteristics in confirmed Saudi breast cancer patients.

## Introduction

Breast cancer (BC) is considered one of the most prevalent cancers among women worldwide, with clinical disparities linked to various molecular subtypes [1,2]. Early detection (ED) of this malignancy is critical for effective treatment and improving survival outcomes [3].

BC is the most prevalent malignant disease in Saudi Arabia (SA), characterized by unique demographic and clinical attributes [4]. The majority of Saudi BC patients are in the age group of 40-59 years, with about two-thirds presenting initially with locally advanced disease [4]. Notably, the incidence of BC in SA has increased at one of the highest rates worldwide, with an average annual percentage change of 5.6%, compared to 0.5-1% in high-income countries. This increase has been associated with stage migration and a rise in the median age at diagnosis [5]. Compared to around 20% of newly diagnosed cases in developed countries, locally advanced and metastatic disease constitutes more than half of new patients with BC in SA. Consequently, this pattern contributes to higher morbidity and mortality rates, along with substantially higher treatment costs [6]. Considering the significant growth in the population of older women and their increased life expectancy, it is essential to prioritize the expansion of mammography screening coverage and the capacity of breast-care services for future planning [5]. Early detection facilitates efficient treatment, resulting in improved patient outcomes and reduced fatality rates [6]. Breast cancer screening, whether conducted through clinical breast examination, breast self-examination (BSE), or mammographic evaluation, is essential for the ED of BC [7]. Therefore, educating women about BC awareness and the importance of regular screening, particularly in age-susceptible groups, constitutes a critical public health priority [7]. A notable proportion of women demonstrate adequate awareness and knowledge of BSE; however, its application is limited, with many individuals uncertain about the correct procedures, timing, and frequency [8]. The low levels of awareness and utilization of mammography underscore the need for targeted educational initiatives to promote ED and improve screening practices [8]. These indicators highlight the necessity of focused educational initiatives and awareness campaigns to address the knowledge deficit and promote ED measures [8]. Our research question emerged from the observed surge in locally advanced and metastatic disease in SA and aims to evaluate the correlation between four demographic variables (age, marital status, employment status, and education) and awareness levels regarding the ED of BC in Jeddah.

## Materials and methods

Study design and setting

This study employed a descriptive cross-sectional model to assess breast cancer symptoms and early-detection awareness among women aged ≥35 years recruited in Jeddah and to examine its association with age, educational attainment, marital status, and employment status. The study was conducted in four shopping malls in Jeddah, SA, during the period from June 1, 2024, to May 30, 2025. The study was initiated by an observed surge in advanced BC among newly diagnosed patients at King Fahad Hospital, Jeddah. This setting was selected because age, gender, and potential risk factors are deemed comparable to those of real patients. The strategic aim was to identify the most vulnerable demographic groups with low levels of awareness regarding the ED of BC to plan focused interventions.

Study participants and sampling

Participants were interviewed by our research assistants in four shopping malls distributed potentially equally around the center of Jeddah. Eligible individuals were selected according to the following inclusion and exclusion criteria by convenience sampling.

Inclusion and exclusion criteria

Inclusion criteria comprised female gender, residency in Jeddah for at least the last six months, and an age of 35 years or older. Exclusion criteria included the absence of any of the three main inclusion criteria (female gender, age over 35 years, and residence in Jeddah for the last six months) and any participant with suspected or confirmed BC.

Data collection tool and technique

A purpose-designed questionnaire was developed and used for data collection. The questionnaire was validated by a panel of ten experts, and all suggested modifications were incorporated to ensure a content validity index of 0.9 or higher. The initial questionnaire was designed in English. The validation was followed by translating the questionnaire into Arabic, and a reliability check was conducted using a pilot study that included 50 female participants of all ages. All designed questions included the option "I did not understand the question" in Arabic. Any question selected as unclear by more than 2% was rephrased, validated, and then underwent a translation reliability check. Any question selected as "I did not understand the question" by more than 5% of the group was considered invalid and removed. An evaluation of 386 responses to the final Arabic questionnaire demonstrated that the 12-item scale, designed to measure various BC clinical presentations, possesses excellent internal consistency (Cronbach's α value = 0.92).

The questionnaire comprised three sections: the first section included the four demographic factors being tested; The second section focused on awareness regarding the common clinical manifestations of BC (breast lump, pain or acute change in size or shape, nipple deformity, retraction or abnormal discharge, skin redness, eczema, dimpling or ulceration, or axillary lump or pain) [9]. The final section included two questions assessing how important ED of BC is perceived to be and the most effective strategy for implementing it (see Appendix, Table 5). Each correct recognition of the 12 signs of BC was assigned one point, and the aggregate score for each participant was then calculated out of the maximum score of 12. The level of awareness was classified into three groups according to the total score for each participant: poor (less than 6 points = <50%), moderate (6-9 points = 50-75%), and good awareness level (10-12 points = >75%) [10].

Sample size and response rate

The sample size was calculated using the Epi Info software (a product of the Centers for Disease Control and Prevention, Atlanta, Georgia, USA). Assuming a 95% confidence level, a 5% margin of error, and a predicted prevalence of 50%, the sample size was estimated at 384 participants. We interviewed 447 participants, of whom 78 were eventually excluded for not meeting one or more of the inclusion criteria. None of the pilot study participants were included in the final analysis. The remaining 369 responses were considered suitable for further analysis of results. 

Data analysis

Data related to this study were initially prepared on a data sheet and subsequently entered into IBM SPSS Statistics Version 22 (IBM Corp., Armonk, NY, USA). Frequencies, percentages, and cross-tabulations were obtained. To evaluate how the four demographic variables correlated with an acceptable level of breast cancer symptom awareness, we used Pearson chi-square tests followed by multinomial logistic regression. For statistical significance, the P-value was set at ≤0.05.

Ethical considerations

The study was approved before the start of data collection by the Institutional Research Review Board of Ibn Sina National College under protocol identification number 017MP08102023. The project was granted the reference number IRRB-01-3112023. The study was conducted anonymously, and the data were only accessible to the research team members. Written informed consent was obtained from each participant in advance. The study protocol adhered to the Declaration of Helsinki of 1975 regarding research on human subjects, as revised in 2000.

## Results

In this study, we reviewed a total of 369 Saudi women, of whom 232 (62.9%) were aged 35-50 years, 94 (25.5%) were aged 50-60 years, and 43 (11.6%) were over the age of 60 years. Out of the studied 369, 255 (69.1%) of the participants were found to have a low level of awareness regarding BC symptoms, while only 35 (9.5%) were found to have a high level of awareness. No statistically significant correlation was observed between participant age groups and their overall awareness of ED determinants for BC (P = 0.532), as shown in Table 1.

**Table 1:** Distribution of the participants by their age-groups aligned with their level of awareness for the ED of BC. BC: breast cancer; ED: early detection.

Variable	35-49 years	50-59 years	60-69 years	70 years and older	Total
Level of awareness of symptoms of BC					
Low	166 (45%)	61 (16.5%)	20 (5.4%)	8 (2.2%)	255 (69.1%)
Moderate	46 (12.5%)	22 (6%)	10 (2.7%)	1 (0.2%)	79 (21.4%)
High	20 (5.4%)	11 (3%)	4 (1.1%)	0 (0.0%)	35 (9.5%)
Total	232 (62.9%)	94 (25.5%)	34 (9.2%)	9 (2.4%)	369 (100%)
Is the ED of BC important in terms of cure and survival rate?					
Not important	22 (6%)	9 (2.4%)	5 (1.4%)	1 (0.2%)	37 (10%)
Important	210 (56.9%)	85 (23%)	29 (7.9%)	8 (2.2%)	332 (90%)
Total	232 (62.9%)	94 (25.5%)	34 (9.2%)	9 (2.4%)	369 (100%)
What is the best method for the ED of BC?					
Don't know	126 (34.1%)	38 (10.3%)	18 (4.9%)	6 (1.6%)	188 (50.9%)
Breast self-exam	48 (13%)	18 (4.9%)	8 (2.2%)	0 (0.0%)	74 (20.1%)
Mammography	58 (15.7%)	38 (10.3%)	8 (2.2%)	3 (0.8%)	107 (29%)
Total	232 (62.9%)	94 (25.5%)	34 (9.2%)	9 (2.4%)	369 (100%)

Regarding the association between the educational attainment of the participants and overall awareness levels, the highest percentage of awareness was among individuals with a university education, followed by those with basic education and high school education, representing 17/153 (11.1%), 10/104 (9.6%), and 8/112 (7.1%), respectively. The results suggest that higher levels of education correlate with an acceptable level of symptomatic awareness necessary for the ED of BC (P-value = 0.004; adjusted odds ratio = 2.55 at a confidence interval of 95%, upper boundary = 4.86, lower boundary = 1.34). The multinomial logistic regression model was constructed using the low awareness level as the reference category for the dependent variables (overall level of awareness for ED of BC), while basic education referenced the independent educational factors and the unmarried group referenced the marital status. The categories for both independent factors (educational attainment and marital status) were entered simultaneously. Despite the relatively small number of participants in the high awareness group, the coefficient B/standard error ratio = 2.8 (>1.96), reflecting good stability of the model and rejecting the possibility of multicollinearity of the independent variables, complete or Quasi-complete separation errors. The multicollinearity diagnostics of this multinomial logistic regression model showed a tolerance of 0.998 and a variance inflation factor of 1.002. Regarding the importance of the ED of BC, most university and higher graduates responded that it is important, followed by those with secondary school and basic education, as shown in Table 2.

**Table 2:** Distribution of participants: educational attainment vs. breast cancer early detection awareness. BC: breast cancer; ED: early detection.

Variable	College	Secondary school	Basic education	Total
Level of awareness of symptoms of BC				
Low	89 (24.1%)	88 (23.8%)	78 (21.1%)	255 (69.1%)
Moderate	47 (12.7%)	16 (4.3%)	16 (4.3%)	79 (21.4%)
High	17 (4.6%)	8 (2.2%)	10 (2.7%)	35 (9.5%)
Total	153 (41.5%)	112 (30.4%)	104 (28.2%)	369 (100%)
Is the ED of BC important in terms of cure and survival rate?				
Not important	4 (1.1%)	16 (4.3%)	17 (4.6%)	37 (10%)
Important	149 (40.4%)	96 (26%)	87 (23.6%)	332 (90%)
Total	153 (41.5%)	112 (30.4%)	104 (28.2%)	369 (100%)
What is the best method for the ED of BC?				
Don't know	64 (17.3%)	56 (15.2%)	68 (18.4%)	188 (50.9%)
Breast self-exam	41 (11.1%)	20 (5.4%)	13 (3.5%)	74 (20.1%)
Mammography	48 (13%)	36 (9.8%)	23 (6.2%)	107 (29%)
Total	153 (41.5%)	112 (30.4%)	104 (28.2%)	369 (100%)

Table 3 summarizes the distribution of participants by their occupation and the aligned level of awareness of ED of BC. All measurements were relatively proportionate. Employment status was not significantly associated with the overall symptomatic awareness of BC (P = 0.287).

**Table 3:** Distribution of participants: employment status vs. breast cancer early detection awareness. BC: breast cancer; ED: early detection.

Variable	Employed	Unemployed	Total
Level of awareness of symptoms of BC			
Low	65 (17.6%)	190 (51.5%)	255 (69.1%)
Moderate	27 (7.3%)	52 (14.1%)	79 (21.4%)
High	11 (3%)	24 (6.5%)	35 (9.5%)
Total	103 (27.9%)	266 (72.1%)	369 (100%)
Is the ED of BC important in terms of cure and survival rate?			
Not important	8 (2.2%)	29 (7.9%)	37 (10%)
Important	95 (25.7%)	237 (64.2%)	332 (90%)
Total	103 (27.9%)	266 (72.1%)	369 (100%)
What is the best method for the ED of BC?			
Don't know	56 (15.2%)	132 (35.8%)	188 (50.9%)
Breast self-exam	12 (3.3%)	62 (16.8%)	74 (20.1%)
Mammography	35 (9.5%)	72 (19.5%)	107 (29%)
Total	103 (27.9%)	266 (72.1%)	369 (100%)

Regarding the marital status of the participants and their aligned level of awareness for ED of BC, high awareness levels were more frequently observed among married individuals, followed by divorced and single individuals, constituting 31/268 (11.6%), 4/69 (5.8%), and 0/32 (0%) in that order (P-value = 0.03, adjusted odds ratio = 3.3 at a confidence interval of 95%, upper boundary = 9.72, lower boundary = 1.125, the coefficient B/standard error ratio = 2.17). Other awareness-related variables were relatively comparable across the three subgroups, as indicated in Table 4.

**Table 4:** Distribution of participants: marital status vs. breast cancer early detection awareness. BC: breast cancer; ED: early detection.

Variable	Married	Divorced	Single	Total
Level of awareness of symptoms of BC				
Low	176 (47.7%)	54 (14.6%)	25 (6.8%)	255 (69.1%)
Moderate	61 (16.5%)	11 (3%)	7 (1.9%)	79 (21.4%)
High	31 (8.4%)	4 (1.1%)	0 (0.0%)	35 (9.5%)
Total	268 (72.6%)	69 (18.7%)	32 (8.7%)	369 (100%)
Is the ED of BC important in terms of cure and survival rate?				
Not important	30 (8.1%)	4 (1.1%)	3 (0.8%)	37 (10%)
Important	238 (64.5%)	65 (17.6%)	29 (7.9%)	332 (90%)
Total	268 (72.6%)	69 (18.7%)	32 (8.7%)	369 (100%)
What is the best method for the ED of BC?				
Don't know	133 (36%)	37 (10%)	18 (4.9%)	188 (50.9%)
Breast self-exam	57 (15.4%)	11 (3%)	6 (1.6%)	74 (20.1%)
Mammography	78 (21.1%)	21 (5.7%)	8 (2.2%)	107 (29%)
Total	268 (72.6%)	69 (18.7%)	32 (8.7%)	369 (100%)

## Discussion

This study evaluated variations in awareness levels of the determinants of ED of BC relative to the underscored sociodemographic factors within the age-susceptible group in SA. The concept was initiated by the clinical observation of an increasing frequency of patients presenting with late-stage BC. Our observation was cross-examined and subsequently supplemented by officially published data [6]. Breast cancer is becoming increasingly common in SA, with rising death rates and challenges in early diagnosis and treatment [11]. Although early diagnosis and advanced treatment have improved survival rates, disparities remain. Nonetheless, the primary objective of this research was to determine whether certain demographic variables correlate with differing levels of awareness regarding the determinants for ED of BC.

The findings indicate that 255 (69.1%) of the participants demonstrated a low level of awareness of the symptoms of breast cancer, with most of them being under the age of 50. The rise in these statistics is primarily attributable to insufficient awareness of symptoms, emotional barriers, and negative perceptions regarding breast screening and health education. These factors collectively contribute to the increasing incidence of clinically advanced BC [11]. Many participants in the current study emphasized the significance of early BC detection, considering mammography to be superior to breast self-examination (BSE). However, awareness of BC symptoms in SA is somewhat mixed; while people have a general understanding of BC, they lack knowledge about specific symptoms and screening methods [12,13]. Hendi et al. reported that about 80% of women in Jazan recognized major warning signs, such as lumps or nipple changes, but demonstrated limited knowledge of BSE techniques and screening mammography [14]. Similar results were reported in other countries in the region, including South Khorasan, Iran, highlighting a consistent pattern of low awareness and insufficient practice regarding ED of BC [15].

Our results suggest that advanced education correlated positively with better recognition of BC symptoms. Previous research within SA mirrors these findings, showing that while general awareness is low, women with college degrees or higher education possess significantly greater knowledge [12]. Similar results were observed in Kenya, likely due to the combined beneficial effects of systematic curricular education and the individual capability and desire to utilize educational resources [16].

Another significant factor in our study was the marital status of the participant; married women showed higher awareness levels towards BC symptoms. Female employees in South Khorasan exhibited generally greater awareness of the ED of BC among married participants compared with single or divorced participants [15]. Evidence indicates that married women experience better breast cancer survival rates, which may be partially driven by increased awareness leading to earlier detection of the disease [17]. Our findings indicate that unlike marital status, employment status did not correlate statistically significantly with the acceptable awareness level required for the ED of BC (P = 0.287). There is growing statistical evidence of a perception in the Middle East and North Africa that social security is better with stable marriage than with labor, particularly in the private sector [18]. Selecting participants based on age susceptibility to BC created a homogeneous group, which likely accounts for the minimal correlation between age and awareness level in our study.

Focused educational campaigns addressing the basic concepts related to the determinants of ED of BC play a significant role in enhancing public awareness. Education level and attendance at BC health campaigns have been identified as significant factors associated with knowledge and attitude scores [19].

The principal limitations of the current study are its cross-sectional design (which cannot infer causality), the potential selection bias from convenience sampling and recruitment in shopping malls, limited generalizability beyond Jeddah, possible interviewer/social-desirability bias, residual confounding, and the small number of participants in the high awareness group. Despite these limitations, the study provides valuable information regarding the correlation between the studied demographic attributes and awareness level for ED of BC in Jeddah, serving as a framework to guide future strategic developments. According to the Saudi Ministry of Health guidelines, the ED of BC has a significant positive impact on clinical outcomes [6]. Our study identified critical gaps in awareness concerning the determinants of early BC detection among at-risk healthy Saudi women. Our results identified two independent demographic attributes as statistically significant correlates of acceptable awareness levels: being married and having a college degree or higher.

## Conclusions

Awareness of BC symptoms and ED methods remains suboptimal among Jeddah inhabitants. Overall awareness of BC symptoms correlates positively with higher levels of education and marital stability. Early detection of breast cancer in Jeddah should prioritize age-susceptible women with low education. It is recommended that future investigations evaluate these demographic attributes in confirmed Saudi breast cancer patients.

## Appendices

**Table 5:** Questionnaire for the study titled the influence of demographic attributes on the awareness level for ED of BC. BC: breast cancer; ED: early detection.

Section No.	Question No.	Question stem	Options
Section (I)	1	Which age category best describes you?	i. 35-49
			ii. 50-59
			iii. 60-69
			iv. 70 and above
	2	What is your marital status?	i. Married
			ii. Widowed
			iii. Divorced
			iv. Single
	3	What is the highest level of education you have completed?	i. High school or equivalent
			ii. Bachelor's degree
			iii. Post-graduate degree
	4	What is your current employment situation?	i. Employed
			ii. Unemployed
Section (II)	5	Do you know any of the signs and symptoms of breast cancer?	i. Yes
			ii. No
	6	Which of the following breast changes can be a sign of breast cancer? (Check all that apply)	i. Lump in the breast
			ii. Lump in the armpit
			iii. Persistent pain in the breast
			iv. Persistent pain in the armpit
			v. New change in the shape or size of the breast
			vi. New change in the position of the nipple
			vii. New pulling inwards of the nipple
			viii. Abnormal nipple discharge
			ix. Breast skin rash or eczema
			x. Breast skin ulceration
			xi. Breast skin redness
			xii. Breast skin dimpling
Section (III)	7	What do you think about the importance of early detection of breast cancer?	i. Not important
			ii. Important to improve treatment outcomes
			iii. I don’t know
	8	Which method do you believe is most effective for detecting breast cancer early? (Mark only ONE option)	i. Mammogram
			ii. Breast self-exam
			iii. I don’t know
